# Exploring the Enhanced Liver Regeneration Patterns Following ALPPS Versus Selective Portal Vein Ligation in an Experimental Model

**DOI:** 10.1002/cnr2.70221

**Published:** 2025-06-04

**Authors:** Dora Krisztina Tihanyi, Attila Szijarto, Andras Fulop, Decan Jiang, Lisa Ernst, Franziska Alexandra Meister, Christian Bleilevens, Alexander Theissen, Henrik Nienhüser, Deniz Uluk, Georg Lurje, Mehrabi Arianeb, Rene H. Tolba, Zoltan Czigany

**Affiliations:** ^1^ Institute for Laboratory Animal Science and Experimental Surgery, Faculty of Medicine RWTH Aachen University Aachen Germany; ^2^ Department of Surgery, Transplantation and Gastroenterology, Hepato‐Pancreatico‐Biliary Surgical Research Center Semmelweis University Budapest Hungary; ^3^ Doctoral School of Clinical Medicine Semmelweis University Budapest Hungary; ^4^ Department of Surgery Campus Charité Mitte|Campus Virchow‐Klinikum–Charité‐Universitätsmedizin Berlin Germany; ^5^ Department of Surgery and Transplantation University Hospital RWTH Aachen Aachen Germany; ^6^ Department of Anesthesiology, Medical Faculty University Hospital RWTH Aachen Aachen Germany; ^7^ Department of General, Viszeral and Transplantation Surgery University Hospital Heidelberg Heidelberg Germany

**Keywords:** ALPPS, animal model, hypertrophy, liver, regeneration

## Abstract

**Background:**

Associating liver partition and portal vein ligation (PVL) for staged hepatectomy (ALPPS) and selective PV embolization (PVE) are important clinical strategies in liver surgery. Even though it has been demonstrated that ALPPS induces a more rapid and expressed hypertrophy than PVL/PVE, this phenomenon is still not well understood.

**Aim:**

In the present study, we aimed to characterize enhanced regeneration patterns in a rat model.

**Methods:**

Male Wistar rats were used (*n* = 84; 220–250 g). Selective PVL and ALPPS were achieved using microsurgical techniques (RML‐regenerating/LML‐non‐regenerating). Parameters of liver regeneration, microcirculation, hepatocyte morphology, hepatocellular injury, and activation status of certain protein kinases involved in liver regeneration were investigated.

**Results:**

Right median lobe (RMLs) in the ALPPS group exhibited a more significant and rapid hypertrophy compared to PVL (regeneration ratio, 1.669 ± 0.155 vs. 1.980 ± 0.189, *p* = 0.009, PVL vs. ALPPS). ALPPS led to a more prominent hepatocellular injury. Hypertrophy was associated with increased microcirculation of the RML and a prominent increase of hepatocellular size (300.43 ± 31.92 μm^2^ vs. 374.48 ± 58.34 μm^2^, PVL vs. ALPPS) and morphology. There was an early pAkt/Akt activation after surgery which was significantly higher in ALPPS (5 ± 2 vs. 9.7 ± 3 RQ‐fold‐change, *p* = 0.0087, PVL vs. ALPPS).

**Conclusions:**

Our results suggest that the enhanced regeneration in ALPPS is associated with characteristic changes in liver microcirculation, cell division, hepatocyte morphology, and activation of pAkt/Akt.

**Abbreviations**
ALPPS: associating liver partition and portal vein ligation
ALT: alanine aminotransferase
ANOVA: analysis of variance
ARRIVE: Animal Research: Reporting of In Vivo Experiments
AST: aspartate aminotransferase
ELISA: enzyme‐linked immunosorbent assay
ERK: extracellular signal regulated kinase
FELASA: Federation of European Laboratory Animal Science Associations
FLR: future liver remnant
GAPDH: glyceraldehyde 3‐phosphate dehydrogenase
IDV: integrated density value
IL‐6: interleukine‐6
ISS: in situ split
JNK: c‐Jun N‐terminal kinases
LANUV NRW: landesamt für natur, umwelt und verbraucherschutz nordrhein‐westfalen
LDH: lactate dehydrogenase
LML: left mediate lobe
O2C: oxygen to see
POD: postoperative day
PVL: portal vein ligation
RML: right mediate lobe
RR: regeneration ratio
SD: standard deviation
SO2: oxygen saturation
TNF: tumor necrosis factor
UK: United Kingdom
USA: United States of America

## 1 Introduction

Radical liver resection remains the best treatment option for liver malignancies [1]. However, approximately 45% of patients have primary unresectable liver tumors due to the inadequate future liver remnant (FLR) [2]. At present, there are several methods that can optimize the volume of the FLR. Selective portal vein ligation (PVL) and portal vein embolization (PVE) are proven techniques to induce hypertrophy of the FLR and permit extended resections [3]. Although these methods have low morbidity, especially PVE has become the clinical standard in many centers, there is a relatively long time interval required to achieve a sufficient hypertrophy of the remnant liver, and in many cases, the overall hypertrophy is not satisfactory or tumor progression in the FLR occurs [4, 5]. Associating liver partition and PVL for staged hepatectomy (ALPPS) was described a decade ago, which combines selective PVL with parenchymal transection [6]. This procedure induces a rapid liver volume increase (40%–150% vs. 20%–50% FLR growth with portal vein occlusion) within a couple of days [6].

The ALPPS procedure was initially described as an accidental finding in the clinical setting and has triggered major clinical attention since its initial report in 2012 [6]. Despite some reverse translational studies which have aimed to describe and characterize this phenomenon of enhanced regeneration and its underlying mechanisms, there are still only a limited number of well‐established animal models and translational studies aiming to investigate the differences behind the enhanced regeneration or hypertrophy triggered by ALPPS versus PVL [7].

Here we describe our microsurgical small animal model of ALPPS versus PVL, focusing on the further characterization of the regeneration response within the first week after both procedures.

## 2 Methods

### 2.1 Animals and Housing

Male Wistar rats weighing 220–250 g (6–9 weeks, *n* = 84, RjHan: WI; Janvier Labs, Le Genest Saint Isle, France) were housed under specific pathogen‐free conditions according to the guidelines of the “Federation for Laboratory Animal Science Associations” (FELASA; www.felasa.eu) with a 12‐h light and dark cycle in a temperature‐ and humidity‐controlled barrier environment. Water and standard rat chow (Sniff GmbH, Soest, Germany) were provided ad libitum. The experiments adhered to institutional guidelines and German federal animal protection laws. The study's ethical proposal received approval from the relevant authorities (LANUV NRW, ID: 84‐02.04.2017.A205). All animals received human care according to the principles of the “Guide for the Care and Use of Laboratory Animals” (8th Edition, NIH Publication, 2011, USA). The present study was designed, performed, and reported according to the principles of the ARRIVE (Animal Research: Reporting of In Vivo Experiments) guidelines [8].

### 2.2 Study Design and Experimental Procedure

**FIGURE 1:**
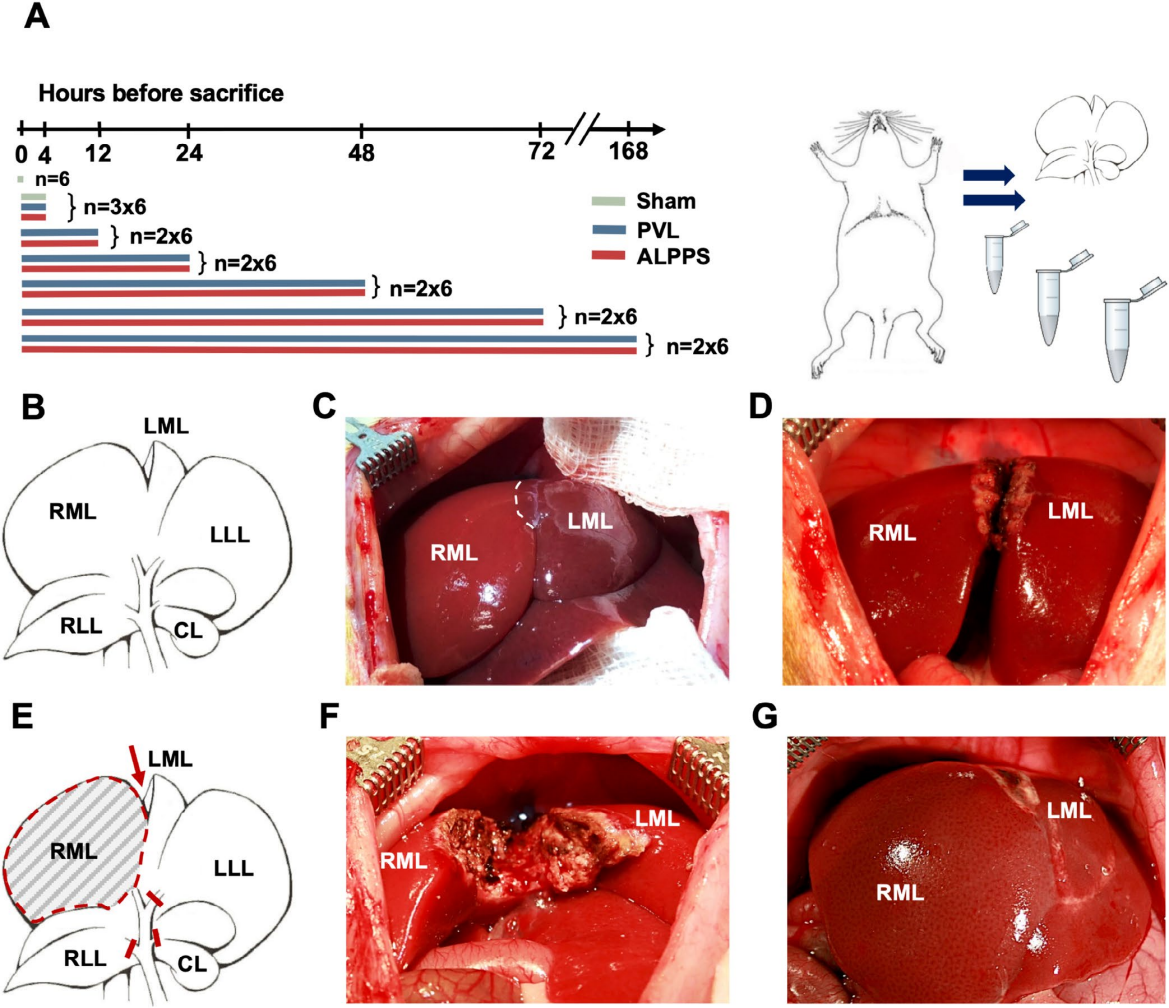
Study flowchart and experimental design. (A) Protocol for sampling and sacrifice. Animals were randomized into three experimental groups: PVL (*n* = 36), ALPPS (*n* = 36), and sham (*n* = 12) operation. Black bars represent the groups according to the different observation periods (*n* = 6/group/timepoint). (B–F) Schematic diagrams (B, E) and photographs (C, D, F, G) of the operative procedure. Portal branches of the caudal lobe (CL), left lateral (LLL), left median (LML), and the right lobe (RL) were ligated. The immediate changes in the circulation of the liver created a visible demarcation line (dashed line) between the portally perfused RML and portally not perfused LML. Parenchymal transection was performed along the falciform ligament. (G) Photo represents the macroscopic changes of the liver lobes on the postoperative 3rd day after parenchymal transection.

Animals were randomized into different experimental groups: selective PVL (*n* = 36), ALPPS (*n* = 36), and sham (*n* = 12) operation (see Figure 1). Sample‐size calculation was used to define the required group sizes. Animals were sacrificed at 7 different time points following the intervention: 0, 4, 12, 24, 48, 72, and 168 h(s) in deep anesthesia. To avoid the disturbing effects of circadian rhythm, all experiments were performed at the same time of day, following an acclimatization period of 1 week. All surgical procedures were performed by the same surgical team (ZC, DKT). Animals were anesthetized with 2 vol% isoflurane (Forane; Abbott GmbH, Wiesbaden, Germany) during all the surgical interventions. Antibiotic treatment and analgesia were achieved by subcutaneous injections of cefuroxime sodium (16 mg/kg/24 h) (Cefuroxim Fresenius; Fresenius Kabi Deutschland GmbH, Bad Homburg, Germany) and buprenorphine (0.03 mg/kg/24 h) (Temgesic; EssexPharma, Haar, Germany) before and for 72 h after surgery. Thereafter, selective PVL was achieved, as described by our group [9, 10, 11]. Figure 1 depicts the main aspect of the surgical procedure. After median laparotomy from the symphysis to the xyphoid process, the median and left lateral lobes were gently mobilized to reveal the portal pedicles. Portal branches of the caudal lobe (CL), left lateral (LLL), left median (LML), and the right lobe (RL) were ligated using 6–0 silk ties (Resorba Medical GmbH, Nürnberg) under preservation of the arterial and biliary branches; meanwhile, the right median lobe (RML) was preserved to regenerate. The preparation was carried out using a surgical microscope (Leica M651 MSD, Leica Mikrosysteme Vertrieb GmbH, Wetzlar, Germany). In the ALPPS group, in situ splitting (ISS) was executed on the median lobe, using a microsurgical bipolar forceps (KLS Martin, Freiburg, Germany) and piercing sutures (Prolene 6–0; Ethicon, Somerville, NJ, USA) [12]. The liver parenchyma was dissected using the bipolar forceps, and larger vascular structures were ligated with sutures on both sides and cut between these ties. The last dorsal approximately 2 mm part of the liver parenchyma to the vena cava was left intact to avoid caval injury, which is almost always associated with death of the animal. Likewise, due to the described super‐selective microsurgical approach, the hepatic vein branches were left intact and were not ligated. Based on our observations, there was no venous congestion (preserved hepatic outflow) and no necrosis of the liver (preserved arterial perfusion) in any of the cases.

In the sham group, the same procedure was implemented without PVL and ISS. At the end of the surgical procedure, the abdominal incision was closed in two layers using continuous 4–0 sutures (Vicryl 4–0; Ethicon, Somerville, NJ, USA). After surgery, the animals were placed in a special cage for intensive care (Vetario; Brinsea Products Ltd., North Somerset, UK) with an oxygen supply and warm air (30°C–35°C) to facilitate recovery from anesthesia. During the follow‐up period, animals were visited and assessed every 12 h, and their clinical state was evaluated blindly using a humane‐endpoints score sheet. As we were interested in the rapid regeneration triggered by PVL + ISS during step I of the ALPPS procedure, partial hepatectomy, described in the clinical nomenclature as ALPPS step II, was omitted in this study. This is in line with the work of other groups (Table 1). There is a vast body of literature describing the characteristics and mechanisms of regeneration after partial hepatectomy in rats [24].

### 2.3 Regeneration Ratio

The wet weight of the liver lobes was measured separately using an analytical scale (AG245; Mettler Toledo, Greifensee, Switzerland). To detect the changes in liver weight, regeneration ratio (RR) was calculated by the following formula: relative liver weight at sacrifice (g/100 g b.w.)/mean of relative liver weight in the control group (g/100 g b.w.).

### 2.4 Ki‐67 Immunohistochemistry

To detect the number of cycling cells, immunostainings were performed for Ki67 (rabbit polyclonal antibody, Abcam) on the regenerating lobes. Tissue samples were fixed in 4% paraformaldehyde for 24 h and embedded in paraffin. Histological slides were scanned (Pannoramic 250 Flash; 3D Histech, Budapest, Hungary). The number of Ki67‐positive hepatocytes was determined by manual counting in 20 random visual fields (200×) for each slide. The investigator performing the evaluation was blinded to the group allocation and to the study protocol.

### 2.5 Biochemical Analysis and Serum Cytokines

Venous blood samples were collected via direct puncture of the inferior vena cava at sacrifice. Samples were centrifuged (4°C, 2 × 10 min, 3000 rpm) and then serum levels of alanine aminotransferase (ALT), aspartate aminotransferase (AST), lactate dehydrogenase (LDH), and total bilirubin levels were measured using standard photometric procedures in an automated analyzer (Vitros 250; Johnson and Johnson, Neuss, Germany).

Serum samples, stored at −80°C, were used for the analysis of the serum levels of tumor necrosis factor alpha (TNF‐alpha) and interleukin‐6 (IL‐6) at 4, 24, 72, and 168 h after index surgery using commercial rat enzyme‐linked immunosorbent assay (ELISA) kits (R and D Systems, Minneapolis, MN, USA) according to the manufacturer's instructions.

### 2.6 Liver Microcirculation

We measured the hepatic microcirculatory perfusion of the LML and RML before and after intervention and directly before sacrifice. The hepatic microcirculation (flow, velocity, and liver surface oxygen saturation‐SO_2_) was evaluated using an O2C device and a corresponding surface probe (O2C‐oxygen to see device, LF1 surface probe; LEA Medizintechnik GmbH, Giessen, Germany). The mean of the measurements from two standard points on each lobe surface was used to characterize microcirculation.

### 2.7 Hepatocyte Size Assessment

Digital images of liver thin sections (3 μm thick) prepared from formalin‐fixed, paraffin‐embedded samples stained by immunofluorescence for b‐catenin (mouse monoclonal antibody, BD Biosciences) were photographed at a magnification of ×400. Hepatocyte size was measured as area in pixels using ImageJ software (Image Processing and Analysis in Java; NIH; http://imagej.nih.gov/), counting 100 hepatocytes per animal manually. Cell surface area was given in μm^2^. The investigator performing the evaluation was blinded from the group allocation and the study protocol.

### 2.8 Protein Expression

Western blotting was carried out as described before [25]. Before Western blotting, liver tissue of the RML was homogenized with ice‐cold lysis buffer (Sigma‐Aldrich, Germany) and Protease inhibitor cocktail tablets (Roche Diagnostics, Mannheim, Germany). Protein concentration in supernatants was determined (DC‐Protein Assay Kit, BIO‐RAD Laboratories, Munich, Germany). Proteins were separated by 10% SDS‐PAGE, and transferred onto a PVDF membrane (BIO‐RAD) according to a standard semi‐dry blotting procedure (60 min, 25 V). Unspecific binding sites were blocked. Incubation with specific antibodies against phosphoinositide 3‐kinase (pi3K), phosphorylated and total pAKT/AKT (protein kinase B), pERK/ERK (extracellular signal‐regulated kinase), pJNK‐p54/JNK‐p54 (c‐Jun N‐terminal kinases), pJNK‐p46/JNK‐p46, and pJNK/JNK (all from Cell Signaling Technology, Danvers MA, USA) was performed. Incubation was followed by repeated washing steps (3 × 5 min in TBS buffer containing 1% Tween20; Sigma‐Aldrich) prior to the incubation with a horseradish peroxidase conjugated goat anti‐rabbit antibody (Cell Signaling Technology) for 1 h at room temperature on a shaker. The final reaction was visualized by enhanced chemiluminescence using an imaging system (Clarity WesternECL Blotting Substrate, ChemiDoc MP System, BIO‐RAD), and the images were analyzed densitometrically using Image Lab Software (BIO‐RAD). The results were displayed as integrated density value (IDV), relative to GAPDH (Glyceraldehyde 3‐phosphate dehydrogenase).

### 2.9 Statistical Analysis

Results are expressed as mean ± standard deviation (SD) for each group, or indicated otherwise. Two‐way analysis of variance (ANOVA) and Bonferroni post hoc correction were performed to analyze changes in time‐dependent parameters and between‐group differences in each timepoint. Depending on the distribution (normal versus not‐normal distribution), one‐way ANOVA or non‐parametric Kruskal–Wallis *H* tests were used to test the differences between three or more groups, while the Student's *t*‐test or Mann–Whitney *U* test were used to investigate the differences within two groups. Differences were considered significant when *p* < 0.05. Calculations and data plotting were performed using IBM SPSS v24 (IBM Inc., Armonk, NY, USA) and GraphPad Prism 9 (GraphPad Software Inc., San Diego, CA, USA) software packages.

## 3 Results

### 3.1 Postoperative Follow‐Up, Survival Rate

Registration of the clinical status was performed every 12 h using a score sheet based on Kanzler et al. as described before [26]. Every animal in the study has survived until the intended observation period. No animal was excluded during analysis.

### 3.2 Liver Regeneration

**FIGURE 2:**
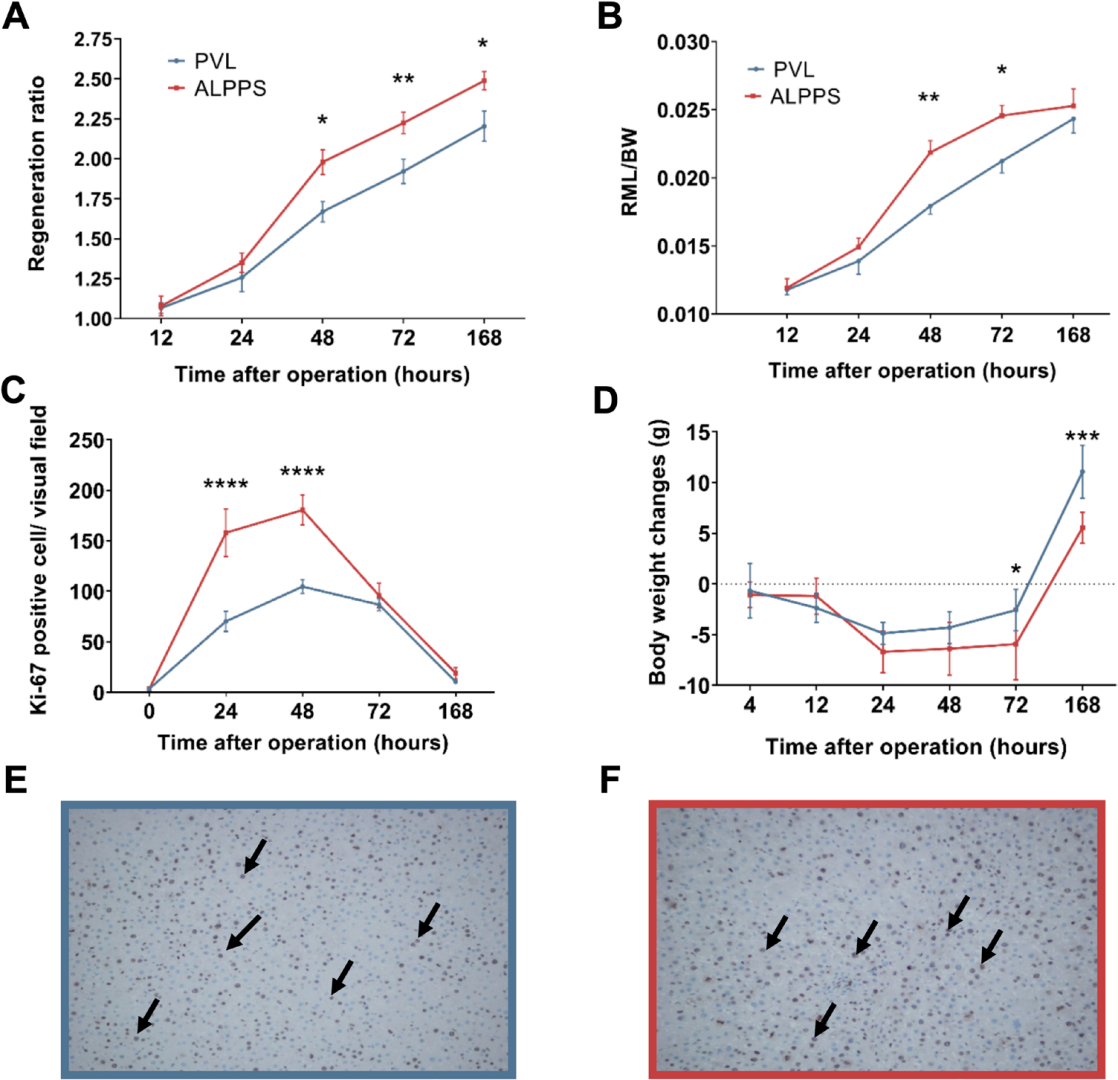
Changes in regeneration rate and cell‐cycle entry. (A) Regeneration rate at the indicated timepoints. Regeneration rate was expressed as a ratio of relative liver weight of RML (g/100 g b.w.) at sacrifice and relative liver weight of RML (g/100 g b.w.) in the control group at 12, 24, 48, 72, and 168 h PVL versus ALPPS. (B) Weight gain of RML in relation to the total body weight (BW) at 12, 24, 48, 72, and 168 h PVL versus ALPPS. (C) Alteration of Ki‐67 index at 24, 48, 72, and 168 h PVL versus ALPPS. (D) Body weight changes at 4, 12, 24, 48, 72, and 168 h PVL versus ALPPS. Values are mean ± standard deviation; *n* = 6/group/timepoint). *p < 0.05, **p < 0.01, ***p < 0.001, ****p < 0.0001 PVL versus ALPPS. (E, F) Representative immunohistochemistry images showing Ki‐67 immunostaining at 48 h. Arrows show Ki‐67 positive cells. Original magnification ×400.

The weight gain of the RML was best reflected by the calculated regeneration ratio (RR). From the 48 h, the RR was significantly higher in the ALPPS group than in the PVL group (postoperative 48 h, 1.669 ± 0.155 vs. 1.980 ± 0.189, *p* = 0.009, PVL vs. ALPPS, Figure 2). Ki‐67 immunohistochemistry showed a marked increase in the counts of cycling cells, which peaks at 48 h, and animals that underwent the ALPPS procedure had significantly higher numbers of dividing cells at 24 and 48 h than in the PVL group (postoperative 24 h, 70 ± 10 vs. 158 ± 24/visual field 200×, *p* < 0.001, PVL vs. ALPPS, postoperative 48 h, 105 ± 6 vs. 180 ± 15 visual field 200×, *p* < 0.001, PVL vs. ALPPS, Figure 2). After the second day, the number of cycling cells decreased to the same extent in both groups (Figure 2).

Parallel to the enhanced regeneration, the body weight of the animals drops below baseline in both groups and recovers rapidly between POD3 and POD7 (Figure 2).

### 3.3 Liver Microcirculation

**FIGURE 3:**
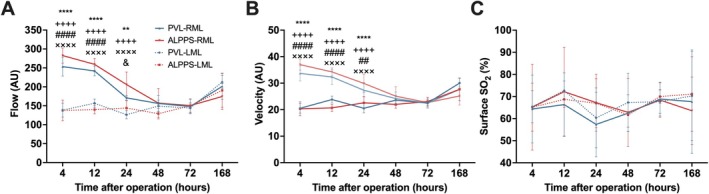
Alterations of liver microcirculation. (A–C) Microcirculatory flow, velocity, and oxygen saturation of RML and LML at 4, 12, 24, 48, 72, and 168 h PVL versus ALPPS. Values are mean ± standard deviation; *n* = 6/group/time point. Continuous lines represent the regenerating RML, while dashed lines show the ligated LML. **p < 0.001, ****p < 0.0001 PVL‐LML versus PVL‐RML, ^++++^ *p* < 0.0001 PVL‐LML versus ALPPS‐RML, ^##^ *p* < 0.01, ^####^ *p* < 0.0001 PVL‐LML versus ALPPS‐RML, ^xxxx^ *p* < 0.0001 ALPPS‐LML versus ALPPS‐RML, ^&^ *p* < 0.05 PVL‐RML versus ALPPS‐RML.

The microcirculatory flow of the ligated LML showed minimal changes compared to the preoperative values (postoperative 4 h, *p* > 0.99, PVL vs. ALPPS, Figure 3). On the further postoperative days, there was no significant change in the portally occluded lobes in either group, and at the end of the week, the liver microcirculatory flow returned near to the baseline values in these lobes (postoperative 168 h, *p* = 0.756 PVL vs. ALPPS, Figure 3). The microcirculatory flow of the portally perfused RML increased after the procedure, with a continuous trend toward higher microcirculation within the first 24 h in the ALPPS group (microcirculatory flow 24 h, 171 ± 6 vs. 206 ± 33, *p* < 0.05, PVL vs. ALPPS, Figure 3). Until the postoperative 7th day, microcirculation returned to the values detected before the operation, and there was no significant difference between the PVL and ALPPS groups (postoperative 168 h, *p* = 0.686, PVL vs. ALPPS, Figure 3).

The measurement of sinusoidal oxygen saturation (SO_2_) on the liver surface demonstrated a large variance between individual animals and did not show conclusive findings throughout the observation period (Figure 3).

### 3.4 Laboratory Blood Tests and Serum Cytokines

**FIGURE 4:**
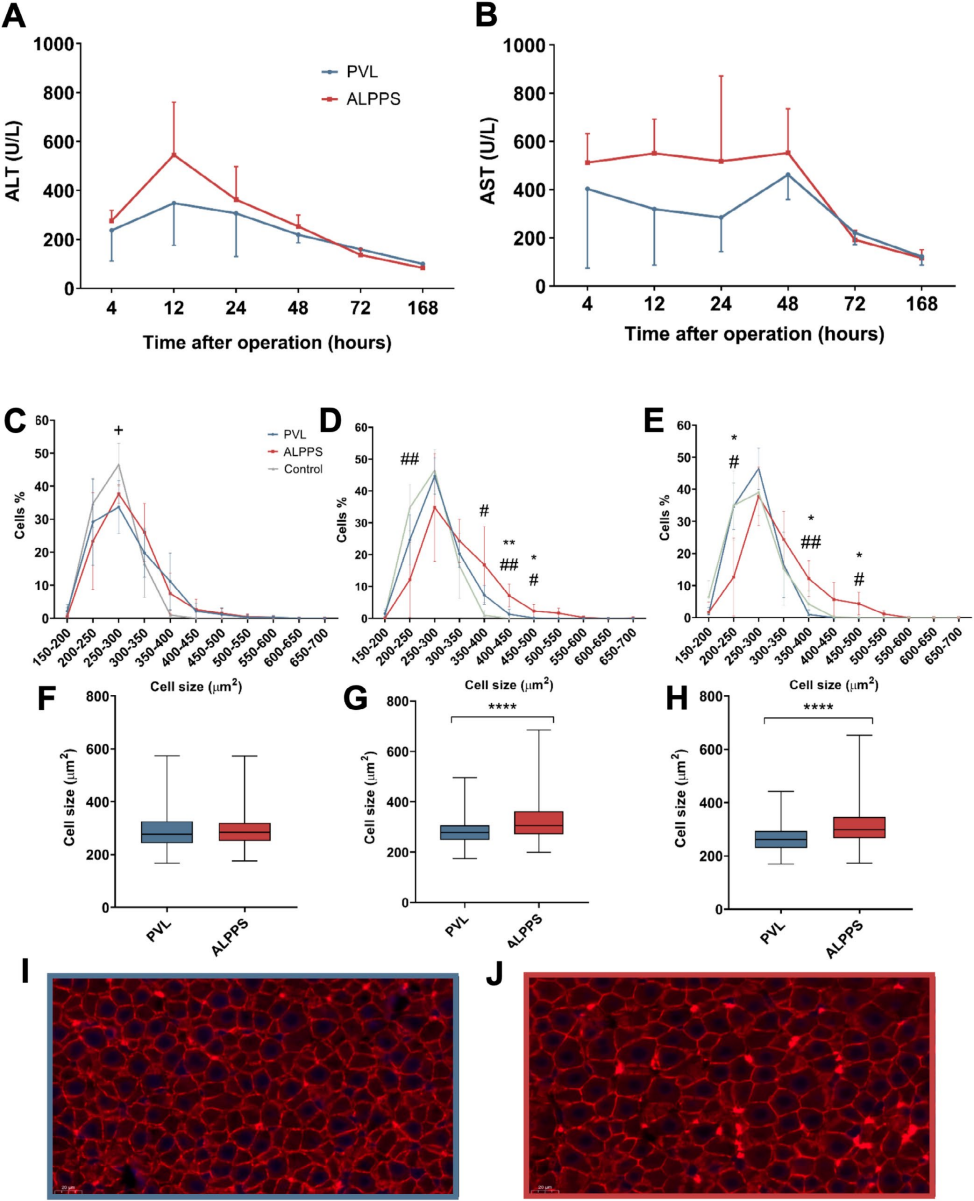
Changes in hepatocyte cell size and morphology. (A, B) Alterations in serum transaminase levels during the observation period at 4, 12, 24, 48, 72, and 168 h PVL versus ALPPS. Values are mean ± standard deviation; *n* = 6/group/time point. (C–E) Percentages of cells by cell size at 48 h (C), 72 h (D), and 168 h (E). ^+^ *p* > 0.05 PVL versus Sham, *p < 0.01, **p < 0.001 PVL versus ALPPS, ^#^ *p* < 0.01, ^##^ *p* < 0.001 ALPPS versus Sham. (F–H) Mean cell size in μm^2^ at 48 h (F), 72 h (G), and 168 h (H), ****p < 0.0001 PVL versus ALPPS. Values are mean ± standard deviation; *n* = 6/group/time point. (I, J) Representative image of beta catenin immunofluorescence staining in PVL (I) and ALPPS (J) at POD3. Original magnification ×400.

No significant alterations were detected regarding the serum albumin and alkaline phosphatase levels (data not shown). However, the concentration of serum transaminases (alanine aminotransferase‐ALT, aspartate aminotransferase‐AST) showed a transient elevation on the first two postoperative days; these values decreased markedly by the end of the week, approaching normal values (Figure 4).

**FIGURE 5:**
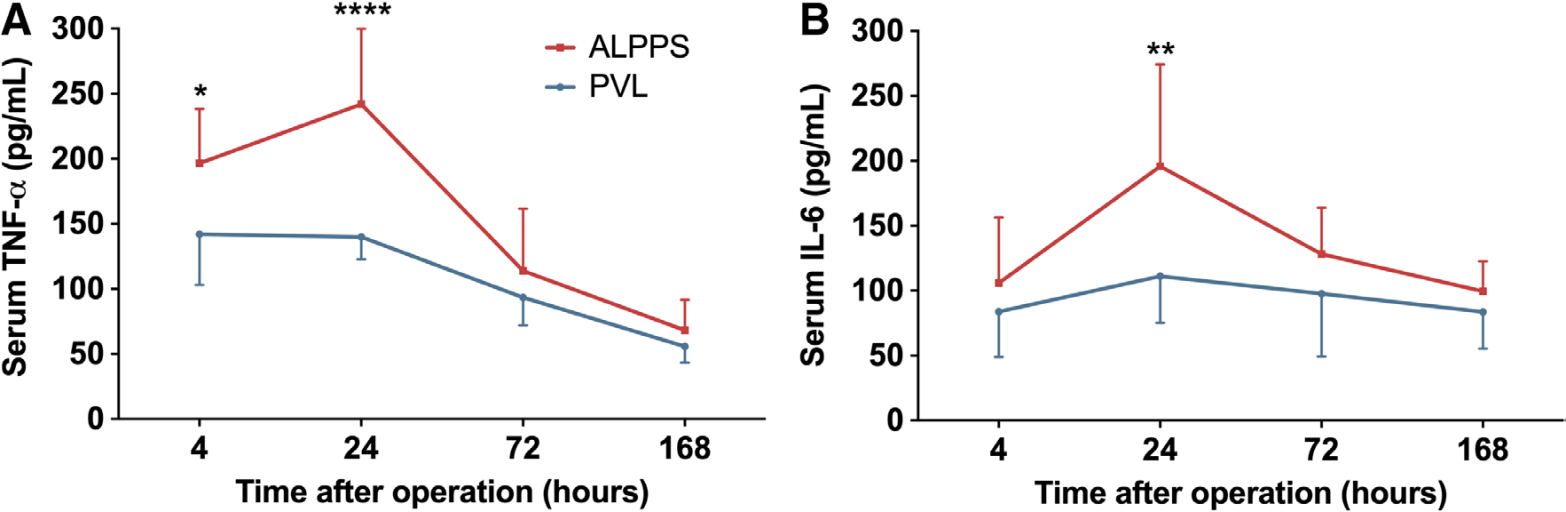
Serum TNF‐alpha and IL‐6 levels. (A, B) Alterations in serum cytokine levels during the observation period at 4, 24, 72, and 168 h PVL versus ALPPS. Values are mean ± standard deviation; *n* = 6/group/time point. *p < 0.01, **p < 0.001, ****p < 0.0001 PVL versus ALPPS.

Serum levels of TNF‐alpha and IL‐6 have peaked 24 h following ALPPS, while flatter curves have been observed in the PVL‐treated animals (Figure 5). At this time point, significantly higher TNF‐alpha and IL‐6 levels were detected in the ALPPS group compared to PVL (serum TNF‐alpha postoperative 4 h, 142 ± 39 vs. 197 ± 42 pg/mL, *p* = 0.0484; serum TNF‐alpha postoperative 24 h, 140 ± 17 vs. 242 ± 58 pg/mL, *p* < 0.0001; serum IL‐6 postoperative 24 h, 111 ± 36 vs. 196 ± 79 pg/mL, *p* = 0.0092, PVL vs. ALPPS, Figure 5). During the later phase of the observation period (72 and 168 h after surgery) both curves have shown similar characteristics without a statistically significant difference between the two groups (Figure 5).

### 3.5 Cell Size and Cellular Hypertrophy

To detect the changes of hepatocyte cell size, b‐catenin immunofluorescence staining was performed. We examined the alterations of the cell size according to the size range on POD2, POD3, and POD7 (Figure 4). Even though only a trend could be observed as early as POD2, on POD3 and POD7, a significantly higher percentage of liver cells were detectable in the larger size ranges, leading to a complete “right shift” in the cell size in ALPPS compared to the PVL group (Figure 4). Accordingly, the overall mean cell size on POD3 and POD7 was significantly higher after ALPPS (postoperative day 3 (POD3), 322.69 ± 34.67 μm^2^ vs. 382.66 ± 55.79 μm^2^, postoperative 7 day (POD7) PVL vs. ALPPS: 300.43 ± 31.92 μm^2^ vs. 374.48 ± 58.34 μm^2^, Figure 4).

### 3.6 Protein Kinases

**FIGURE 6:**
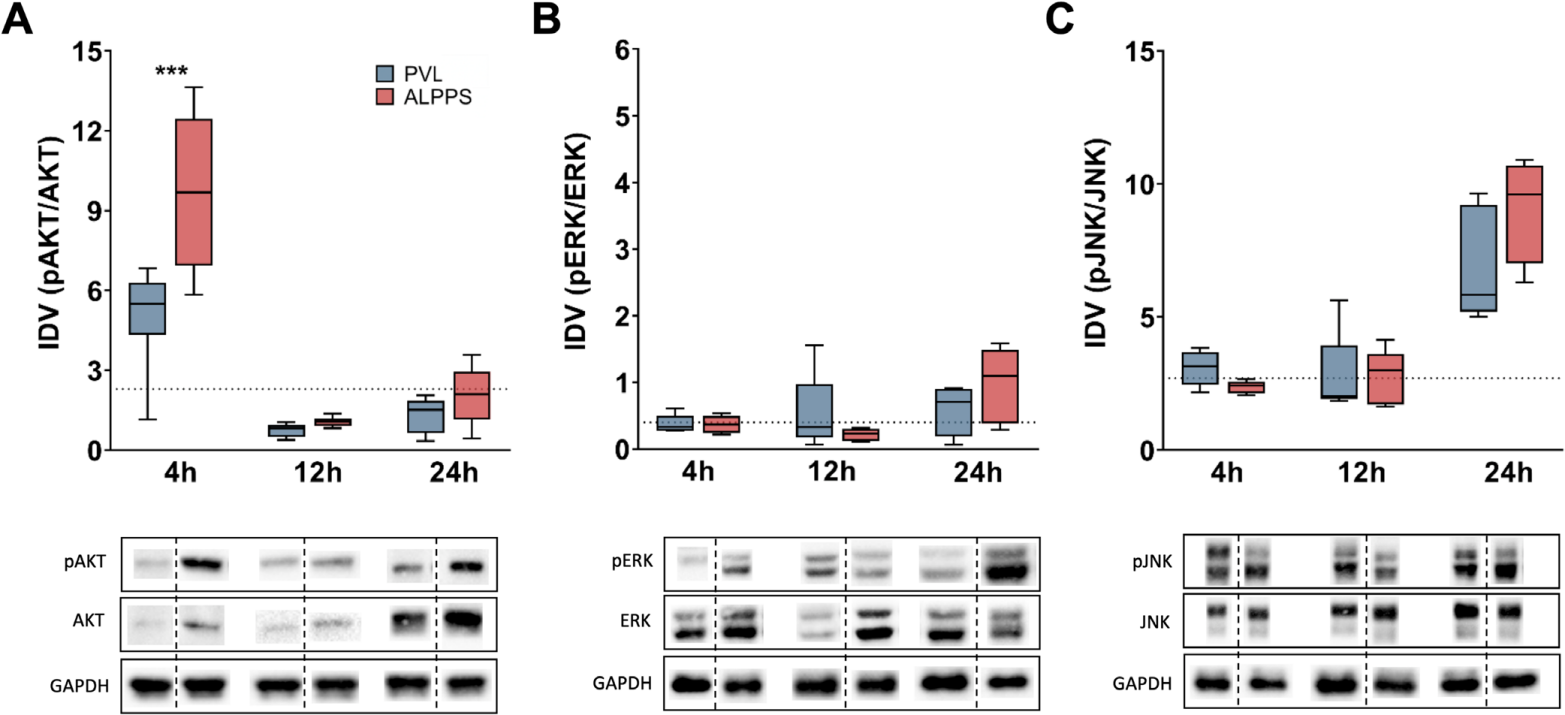
Tissue protein levels pAKT/Akt, pERK/ERK, pJNK/JNK. (A–C) Relative protein expression of pAKT/Akt, pERK/ERK, pJNK/JNK in liver tissue at 4, 12, 24 h PVL versus ALPPS, ***p < 0.001. Values are mean ± standard deviation; *n* = 6/group/time point. The vertical dotted lines in the Western blot images denote that the blots have been spliced to remove the triplicate lanes. Western blot analysis shown is representative of independent experiments. Complete raw data has been provided in the online.

Liver tissue protein levels (RML) of various elements of the mitogen‐activated protein kinase and pi3K/Akt pathways were analyzed using Western blotting. Here, besides some non‐characteristic fluctuations over the baseline (Figures 6 and S1), a marked pAkt/Akt activation was observed as early as 4 h in both experimental groups. This peak was found to be significantly higher in the liver of the animals which underwent the ALPPS procedure compared to PVL (4 h PVL vs. ALPPS: 5 ± 2 vs. 9.7 ± 3 RQ fold change, *p* = 0.0087, Figure 6).

## 4 Discussion

In the present study, we explore and describe some important differences of the enhanced liver regeneration triggered by ALPPS versus selective PVL in our well‐established and reproducible small animal model using rats. We show that the enhanced liver regeneration triggered by ALPPS is associated with characteristic changes in liver microcirculation, cell size and morphology, increased serum levels of proliferative cytokines, and associated activation of pAkt/Akt.

Liver regeneration response and enhanced hypertrophy in ALPPS have been investigated in different experimental and clinical studies and publications over the past years [21, 27, 28]. However, consistent data from well‐designed animal studies are still relatively rare [7]. We have summarized a selection of the most impactful models in Table 1. Our present model features a fine preparation technique using a surgical microscope, energy tools (bipolar microforceps), and piecing sutures. These sophisticated surgical techniques with the high standards of our animal and microsurgical laboratory facilities (certification of quality assurance according to the Deutsches Institut fur Normung/German Institute for Standardisation; International Standardization Organization; DIN: ISO9001:2015, Biosafety Level‐1 microsurgical lab) ensured the validity of our data and the reproducibility of the model.

**TABLE 1:** A selection of rodent models of ALPPS and their main variations*.

First author, year of publication	Species	Ligated lobes	FLR lobe	Step I procedures	Step II y/n	Proliferation peak
Schlegel et al. 2014 [13]	Mouse	RML, RL, CL	LML	PVL, ISS (bipolar), PHx 30%	Y	POD4
Yao et al. 2014 [14]	Rat	LLL, LML, RL, CL	RML	PVL, ISS (ligation, microtweezers)	N	POD2
Almau Trenard et al. 2014 [15]	Rat	LLL, LML, RL, CL	RML	PVL, ISS (U‐stitches, scissors)	N	n.r.
Dhar et al. 2015 [16]	Rat	LLL, LML, RL, CL	RML	PVL, ISS (pringle, sutures)	N	POD2
Shi et al. 2015 [17]	Rat	LLL, RML, RL, CL	LML	PVL, ISS (disposable drip bipolar forceps), PHx 30%	Y	POD2
Garcia Perez et al. 2015 [18]	Rat	LLL, RML, RL	LML + CL	PVL, ISS (bipolar forceps)	Y	POD2
Wei et al. 2016 [19]	Rat	LLL, LML, RL	RML	PVL, ISS (clamp and piercieng sutures), PHx 30%	Y	POD1
Kawaguchi et al. 2017 [20]	Rat	LLL, LML, RL	RML	PVL, ISS (bipolar scissors), LHV ligation	N	POD2
Schadde et al. 2017 [21]	Rat	LLL, LML, RL, CL	RML	PVL, ISS (bipolar forceps, scissors)	N	n.r.
Sheng et al. 2017 [22]	Rat	LLL, LML, RL, CL	RML	PVL, ISS (clamp and sutures)	N	n.r.
Tong et al. 2018 [23]	Rat	LLL, LML, RL, CL	RML	PVL, ISS (clamp and ligatures)	Y	POD2
Budai et al. 2018 and Tihanyi et al. 2024 (present report) [11]	Rat	LLL, LML, RL, CL	RML	PVL, ISS (microscopic prep, bipolar, piercing sutures)	N	POD1‐2

Abbreviations: CL, caudate lobe; FLR, future liver remnant; ISS, in situ split; LLL, left lateral lobe; LML, left medial lobe; PHx, partial hepatectomy; POD, postoperative day; PVL, portal vein ligation; RL, right lobe; RML‐right medial lobe.

* This selection of reports is not all‐comprehensive. It is based on a literature search without the stringent criteria of a systematic review; studies where the authors declared that they used a certain model described by another group before were omitted (PubMed, search date, 04‐07‐2024).

In this model, the enhanced regeneration triggered by ALPPS was clearly observed from POD2, where the RR and RML to body weight ratio curves of the ALPPS group started to show a steeper slope compared to those of the PVL group. Parallel to this, Ki‐67 immunohistochemistry showed a markedly higher number of cycling cells in the ALPPS group. These findings are in line with previous observations of our group and those of others [7, 9, 10, 28, 29, 30, 31], confirming the reliability and reproducibility of our model. Even though all animals have survived the observation period until sacrifice in excellent clinical condition, the distinctly higher serum transaminases and more pronounced body weight loss in the animals of the ALPPS group suggested a more severe surgical trauma in the groups where in situ split was an integral part of the procedure. In line with this, clinical cohorts often described a higher morbidity of ALPPS compared to other less invasive FLR augmentation techniques [32].

Cytokines such as TNF‐α and IL‐6 are important promoters of liver regeneration [18, 33, 34]. These cytokines and other growth factors were found to be increased in the serum of patients undergoing ALPPS [33]. In line with these findings, we could detect significantly increased TNF‐alpha and IL‐6 levels in the samples of ALPPS‐treated animals compared to PVL in our model. Even though these alterations are not specific for ALPPS and can also be observed after conventional partial hepatectomies, the distinct differences in the kinetic changes determine the enhanced hypertrophy/regeneration observed following ALPPS compared to PVL [33]. It should be noted, however, that besides their important role as promoters of liver regeneration, TNF‐α and IL‐6 are both showing dichotomous negative effects in the liver, such as promoting cell death, liver toxicity, inflammation, and liver aging, fibrosis, steatosis, respectively [35, 36].

Alterations of the macro‐ and microcirculation following ALPPS, selective PVL, or partial hepatectomy have been suggested to be a primary and very early stimulus in inducing liver regeneration, often called the “blood flow theory” [31]. Upon PVL in our model, RML remains the only part of the liver with retained portal venous perfusion, which thereby exposes FLR, in particular the sinusoidal endothelium and Kupffer cells, to extensive hemodynamic forces [37]. This increased sheer stress triggers a number of intracellular events such as the activation of cell cycle‐associated genes and transcription factors [37]. We monitored liver microcirculation flow, velocity, and SO_2_ with an O2C device. Accordingly, the microcirculation of the RML increased substantially over the first days in PVL and even more in ALPPS, which confirms the potential role of microcirculation as an early driver of hypertrophy in this model [37, 38]. No major changes were recorded in the liver lobes with selectively ligated PV branches. This latter phenomenon might be attributed to the hepatic artery buffer response, where a subsequent increase of hepatic arterial flow can buffer the portal flow change in the ligated lobes [37]. The above‐mentioned “blood flow theory” is also exploited by the relatively new approach of liver venous deprivation which also results in a more expressed hypertrophy compared to PVE, probably via hemdynamic changes and increased blood flow in the contralateral liver by closing not just the portal vein branches but also the hepatic veins [39].

Macroscopically, total cessation of portal venous inflow in the ligated lobes leads to atrophy, compensated by a volume and weight gain of the non‐ligated RML, depicted well by our Figure 1. This results in a more or less constant liver volume and weight [30]. At this point, it is important to differentiate between hepatocyte proliferation and hypertrophy in FLR augmentation techniques such as ALPPS and PVL [40]. However, the distribution between the two mechanisms of liver growth is individual and depends on the model and other cofactors [41]. As discussed above, we could observe a markedly higher rate of hepatocyte proliferation in the ALPPS group compared to PVL in our model. Hypertrophy is a mechanism that allows rapid volume increase of the liver without generating new cells. In a previous study, we found that at the end of the regeneration plateau, non‐ligated lobes are characterized by enlarged hepatic lobules [30]. Nevertheless, there is only limited data in the setting of ALPPS versus PVL on structural changes of the hepatocyte morphology itself. In a small clinical series of 8 ALPPS versus 14 PVL patients, Matsuo et al. have demonstrated greater hepatocyte density and smaller hepatocyte size in ALPPS than in PVE, which is in contrast to our findings [42]. However, almost all patients in this clinical trial underwent intensive neoadjuvant chemotherapy, which has probably influenced the results on hepatocyte morphology. We show an increase in the average hepatocyte size and a “right shift” toward larger cell size categories in the ALPPS animals. This is in line with the data reported by Sheng et al., also showing larger hepatocyte size following ALPPS, even though the authors arrive at this conclusion by counting only 50 cells per animal [22]. Unfortunately, functional assessment of the liver was beyond the scope of our present study; based on previous findings of our group and those of others [22], we strongly assume that these larger hypertrophic hepatocytes are functionally immature, leading to a discrepancy between size/volume and function of the RML [11]. This, together with the major surgical stress, might partially explain the higher morbidity following ALPPS that was observed in some clinical patient cohorts and represents one of the most controversial and critical issues around the use of this procedure [11, 32].

Mitogen‐activated protein kinase as well as the pi3K/Akt/mTOR pathways are involved in various cellular functions as a downstream effector of growth factors, regulating cell proliferation and survival; therefore, they are considered key pathways in liver regeneration [43]. Among their roles, there are regulatory functions of the cell cycle, growth, and metabolism [43]. In line with this, we could observe a strong activation of pAkt/Akt in the early hours after surgery, with values almost 10‐fold over the baseline for ALPPS versus 5‐fold in the PVL group. In the past, concerns have been raised that the activation of these pro‐survival proliferation cascades and the associated rapid hypertrophy of the liver might lead to tumor progression after ALPPS [33]. Although this topic is still in part controversially discussed, an elegant experimental study by Kambakamba et al. showed that ALPPS does not appear to enhance the growth of colorectal metastases [44].

Certain limitations apply to this experimental study, which need to be reported here. We acknowledge the descriptive nature of our study, where we could not reveal deep mechanistic aspects underlying enhanced hypertrophy triggered by ALPPS, which overall limits the novelty of our study. With a limited number of analysis timepoints and the lack of a repetitive sample collection from the individual animals (due to 3R considerations to limit severity and stress) we may have missed the critical window, e.g., in the activation of various kinases, leading to some non‐conclusive findings. It was beyond the scope of the present exploratory study to deliver further comprehensive mechanistic data, which would have required carrying out extensive in vivo and in vitro studies with specific inhibitors, gene expression analyses, and transgenic strains; even though these experiments would have significantly increased the value of our data and the impact of our study. The clinical relevance of this study is also slightly limited by the use of PVL instead of PVE as control, which represents the clinical reality in most liver surgical units. Nevertheless, this was based on pragmatic reasons, as PVE is technically more difficult to establish and less reproducible in small rodents. Based on these limitations, our data can only be interpreted with caution.

## 5 Conclusions

Notwithstanding these limitations, this is a meticulously executed experimental study using a reproducible animal model and high reporting standard as well as data integrity, showing novel findings on cell morphology and microcirculation in ALPPS versus PVL. There are confirmatory findings and additional data enriching the literature on experimental ALPPS and PVL. Future studies with a direct translational aspect including human subjects and in‐depth functional and molecular analyses are warranted to further extend our knowledge on the ALPPS‐related enhanced liver regeneration.

## Author Contributions

The study was designed by the initiating study team (D.K.T., Z.C., R.H.T., A.S., and A.F.). Experiments, data collection, analysis, and interpretation were performed by Z.C., D.K.T., R.H.T., C.B., A.T., H.N., and A.M. The manuscript was drafted by Z.C., and D.K.T. All authors (D.J., L.E., F.A.M., H.N., D.U., G.L., and A.M.) have substantially contributed to the overall analysis plan, interpreting interim and final results, and critically revising and approving the final version of the manuscript.

## Ethics Statement

The ethical proposal of the study was approved by the responsible authorities (LANUV NRW, ID: 84‐02.04.2017.A205). All animals received human care according to the principles of the “Guide for the Care and Use of Laboratory Animals” (8th Edition, NIH Publication, 2011, USA). The current study was conceived, carried out and reported according to the main principles of the ARRIVE (Animal Research: Reporting of In Vivo Experiments) guidelines [8].

## Conflicts of Interest

The authors declare no conflicts of interest.

## Supporting information


**Figure S1.** Tissue protein levels pi3K, pJNK‐p54/pJNK‐p54, pJNK‐p46/JNK‐p46. (A–C) Relative protein expression of pJNK‐p54/pJNK‐p54, pJNK‐p46/JNK‐p46in liver tissue at 4, 12, 24 h PVL versus ALPPS, Values are mean ± standard deviation; *n* = 6/group/timepoint.

## Data Availability

All relevant data were reported within the paper and/or in the online Supporting Information. Further supporting data will be provided upon written request addressed to the corresponding author.
