# Supplementary material for: Exploring the Enhanced Liver Regeneration Patterns Following ALPPS Versus Selective Portal Vein Ligation in an Experimental Model
Source: Cancer Rep (Hoboken). 2025 Jun 4;8(6):e70221. doi: 10.1002/cnr2.70221 (PMC12134493; doi:10.1002/cnr2.70221)
Supplement: Supplementary file 1 — Figure S1. Tissue protein levels pi3K, pJNK‐p54/pJNK‐p54, pJNK‐p46/JNK‐p46. (A–C) Relative protein expression of pJNK‐p54/pJNK‐p54, pJNK‐p46/JNK‐p46in liver tissue at 4, 12, 24 h PVL versus ALPPS, Values are mean ± standard deviation; n = 6/group/timepoint. [file CNR2-8-e70221-s001.pdf]

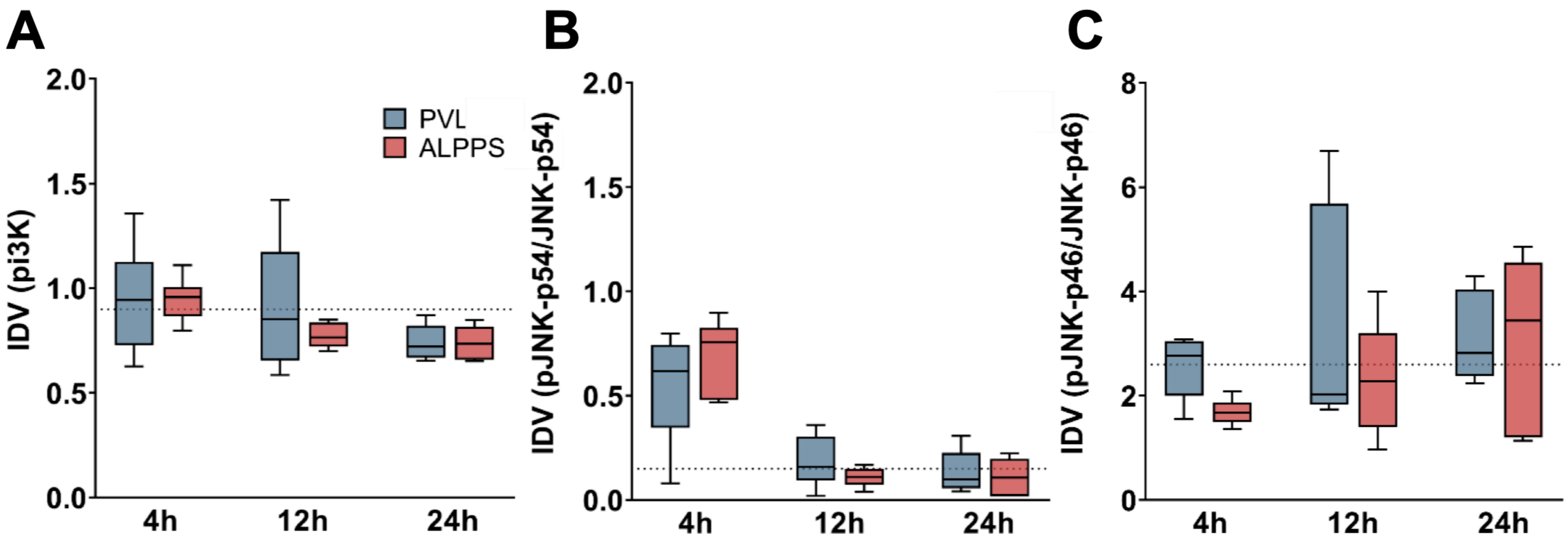

Figure S1: Tissue protein levels pi3K, pJNK-p54/pJNK-p54, pJNK-p46/JNK-p46
(A-C) Relative protein expression of pJNK-p54/pJNK-p54, pJNK-p46/JNK-p46in liver tissue at 4,12, 24 h PVL versus ALPPS, Values are mean ± standard deviation; n=6/group/timepoint).
